# Pregnancy-Related Maternal Risk Factors of Attention-Deficit Hyperactivity Disorder: A Case-Control Study

**DOI:** 10.5402/2012/458064

**Published:** 2012-06-05

**Authors:** Shahrokh Amiri, Ayyoub Malek, Majid Sadegfard, Salman Abdi

**Affiliations:** ^1^Department of Psychiatry, Clinical Psychiatry Research Center (CPRC), Tabriz University of Medical Sciences, Tabriz, Iran; ^2^Department of Psychiatry, Razi Mental Hospital, El Goli Boulevard, P.O. Box 5456, Tabriz 5167846184, Iran; ^3^Department of Psychiatry, Tabriz University of Medical Sciences, Tabriz, Iran; ^4^Department of Psychology, Tabriz University of Medical Sciences, Tabriz, Iran

## Abstract

*Background*. The etiology of attention-deficit hyperactivity disorder (ADHD) is complex.This study was conducted to evaluate the pregnancy-related maternal risk factors of ADHD. *Methods*. 164 ADHD children attending to Child and Adolescent Psychiatric Clinics compared with 166 normal children selected in a random-cluster method from primary schools. ADHD rating scale and clinical interview based on Schedule for Affective disorders and Schizophrenia for School-Aged Children (K-SADS) were used to diagnose ADHD cases and to select the control group. *Results*. The mean maternal age at pregnancy, duration of pregnancy, and the mean paternal age were alike in two groups. The ADHD children's mothers compared with those of control group had higher frequencies of somatic diseases, psychiatric disorders, and alcohol and cigarette exposure during the pregnancies (*P* < 0.01). Also birth by cesarean section was more common among mothers of ADHD children (*P* < 0.001). These factors plus trauma to the abdomen during pregnancy were significantly predictors of ADHD in children. *Conclusions*. Some pregnancy-related maternal factors may be considered as environmental risk factors for ADHD. Each of these factors considered in our study as a risk factor needs to be tested and confirmed through next methodologically appropriate researches in this field.

## 1. Introduction

Attention-deficit hyperactivity disorder (ADHD) is one of the most common psychiatric disorders among school-aged children. Reports on the prevalence of ADHD in the United States have varied from 2 to 20 percent of grade-school children [1]. In a study in Brazil, the prevalence of ADHD in children of 4 primary schools was 13% with a higher rate among boys in comparison to the girls [2]. The prevalence of ADHD in primary school children in Tabriz, Iran, has been reported to be 9.7% [3].

Regarding to the high prevalence of ADHD and its psychosocial outcomes such as social isolation and stigma, negative attributions by the peers, conduct disorder, substance-related disorders, and mood disorders, the importance of preventive and therapeutic interventions is revealed. However, as the etiology of ADHD is complex and most likely includes genetic and environmental factors, preventive and therapeutic interventions may not be sufficient unless we could discover much more factors involved in developing ADHD. Hence, considering the etiological factors with different approaches is something of importance. As it is shown, genetic predisposition is a major cause in ADHD, but it does not follow the Mendelian patterns of inheritance and it is likely to be a polygenetic disorder so that genes can exert their influence only via interaction with the environment. On the other hand, the pathogenetic mechanisms for expression of various genes in interaction with various environmental factors can vary widely from one individual to another [4]. This issue emphasizes on the need to study and discovering much more environmental factors as risk factors for ADHD through appropriate research designs.

A number of nongenetic environmental factors have been shown to be associated with ADHD. Some of these factors include traumatic brain injury [5, 6], exposure to environmental toxins such as zinc or lead [7], very severe early deprivation [8], greater levels of environmental adversity including low social class, maternal psychopathology, and family conflict [9, 10].

Review of the literature shows that some of pregnancy-related factors also may be effective in children's health. Unwanted pregnancy that may be followed with elective abortion may have major negative psychological and physical outcomes. Some studies have reported the association of unwanted pregnancies with neonatal mortality, childhood schizophrenia, and child abuse [11–14]. However, regarding the cultural psychiatry, the unwanted pregnancy, and elective abortion may have different negative outcomes in various cultures.

Among other factors, also, exposure to cigarette during pregnancy has been shown to be a risk factor for ADHD and conduct disorder [15–18]. It has been demonstrated that even low doses of alcohol may also lead to later difficulties with attention and behavior [19, 20].

In Parent Management Training (PMT) sessions for mothers of ADHD children, held in our department, we were asked different questions about the probable pregnancy-related maternal causes of ADHD. As the literature lacked to give us the definite answers to their questions, so this matter was the main motive for us to design a research to study the probable role of some pregnancy-related events as risk factors for ADHD. Hence, we hypothesized that different pregnancy-related factors or events may be some of contributing factors for ADHD. These factors included: the mean age of parents at pregnancy, duration of pregnancy, recognized somatic disease or psychiatric disorder, cigarette and alcohol exposure, cesarean section, X-ray exposure, trauma to the abdomen (severe enough to be the focus of clinical attention), infections, dysentery, bleeding, preeclampsia, unwanted pregnancy, and attempted abortion.

## 2. Methods

### 2.1. Sample and Procedure

This case-control study was performed in 2009. 164 children with ADHD were selected with a nonrandom convenience method from sequential referrals to child and adolescent psychiatric clinics of Tabriz University of Medical Sciences, Iran. Also 166 healthy students were selected as control groups.

 Regarding the age range of the case group, the control group was selected with a random cluster sampling method from among primary schools' students in Tabriz, Iran. They were recruited from a concurrent epidemiological study done among the primary school's students [3]. There were no cases to refuse the study both in case and control samples. Based on the approval of the project by the medical ethics committee of the Tabriz University of Medical Sciences and after obtaining the written informed consent from participants, data collection was performed via an interview with and history taking from mothers by psychiatrist, using a check list including pregnancy-related maternal factors. The mothers were asked to confirm or not the presence of the above-mentioned events during their pregnancies. To avoid the recall bias, whenever it was possible, the medical documents of the mothers were reviewed to confirm the presence of the event and increase the reliability of the answers. Then the frequencies of these events during pregnancy were compared between case and control groups.

### 2.2. Inclusion and Exclusion Criteria

Inclusion criteria for case group were clinical diagnosis of ADHD according to DSM-IV-TR and parental written consent. The children with general somatic diseases and major psychiatric comorbidities were excluded. Inclusion criteria for control group were diagnosis of “no major psychiatric disorders” and parental written consent. The children with general somatic diseases were excluded.

### 2.3. Measures

#### 2.3.1. ADHD Rating Scale—Parent Version

The ADHD rating scale includes 18 items that each one shows one ADHD symptom according to DSM-IV-TR criteria. It may be used for age range of five to 18 years and is useful for differentiation of ADHD and healthy children and differentiate attention-deficit symptoms from hyperactivity and impulsivity symptoms. The validity of ADHD rating scale is approved by Dupaul et al., and its reliability is reported to be high [21]. We used this questionnaire for primary diagnosis of ADHD among case group. 

#### 2.3.2. Schedule for Affective Disorders and Schizophrenia for School-Aged Children (K-SADS)

This is a diagnostic semistructural interview designed according to DSM-III-R and DSM-IV and was filled via an interview with parents and children by a psychiatrist. The reliability of Persian version of K-SADS diagnostic interview was reported to be 0.81 in test-retest method and 0.69 in interrater reliability by Ghanizadeh et al. in Iran [22]. They reported a high sensitivity and specificity for Farsi version of K-SADS instrument. We used this tool for diagnosis of ADHD and psychiatric comorbidity in ADHD group and checking for healthy psychiatric status in control group. 

### 2.4. Data Analysis

The obtained data were analyzed by descriptive methods (mean, standard deviation, frequency, and percent) and Fisher's exact test. Also for determining the contributing factors for ADHD, the logistic regression analysis was used. Data were analyzed using SPSS (version 17) software (Statistical Procedures for Social Sciences; Chicago, IL, USA), and *P* value less than 0.05 was considered statistically significant.

## 3. Results

Mean age of the children was 9.2 ± 2.23 years and 9.02 ± 1.53 years, in case and control groups, respectively. According to Table 1, the obtained results from independent-sample *t*-test showed no statistically significant difference between mean parental ages at pregnancy and pregnancy duration in case and control groups.

According to Table 2, the ADHD children's mothers compared with those of control group had higher frequencies of somatic diseases, psychiatric disorders, and alcohol and cigarette exposure during the pregnancies (*P* < 0.01). Also they gave more birth by cesarean section (*P* < 0.001). The frequency of trauma to the abdomen, X-ray exposure, infections, dysentery, bleeding, preeclampsia, unwanted pregnancy, and attempted abortion was not significantly differed between the case and control groups.

The results obtained from binary logistic regression test showed that somatic disease (*P* = 0.04), psychiatric disorder (*P* = 0.008), trauma to the abdomen (*P* = 0.04), cigarette and alcohol exposure (*P* = 0.023), and cesarean section (*P* < 0.001) in mothers were predictors of ADHD in children.

## 4. Discussion

The results of our study showed that the parental age at pregnancy had no effect on developing ADHD. Our findings were not similar to the results of a study conducted by Valdimarsdóttir et al. that showed that low maternal age is related to ADHD in neonates [23]. Since the results of the study done by Ghanizadeh. showed no association between birth order and ADHD [24], it may be concluded that parental age in children with ADHD is not higher than that of healthy children. However, the association of high parental especially paternal age and some psychiatric problems has been revealed [25]. But, at the moment, there are no biological evidences to show the association between ADHD and parental age at pregnancy.

Results obtained in current study about the role of exposure to alcohol and cigarette during pregnancy are similar to those reported by some authors [15, 16, 23]. Essentially, the fetal exposure to nicotine is related to different behavioral and neurological outcomes such as cognitive disorders, ADHD, and other psychiatric disorders [26]. As mentioned in the Introduction of this paper it has been demonstrated that even low doses of alcohol may also lead to later difficulties with attention and behavior [19, 20]. In general, the maternal life style may be a risk factor for ADHD and related symptoms [27]. 

According to our findings, birth by cesarean section is a risk factor for ADHD in children. Our results are similar to those previously reported by Valdimarsdóttir et al. [23]. Having a cesarean section rather than a vaginal birth may have psychological effects on women and their ability to adjust to motherhood. Some studies indicate that a caesarean birth, especially one that was not anticipated, may increase the risk of depression and posttraumatic stress in some women [28, 29]. It seems that cesarean section due to its psychological impacts on mothers may affect maternal attitudes and behaviors in relation to their children and in turn result in an increase in symptom expression of ADHD in offspring. This is a psychological interpretation based on the previous studies, but it should be considered that cesarean section may not be independent at birth from the brain status of the newborn.

The results showed that suffering from diseases (both somatic diseases and psychiatric disorders) during pregnancy have a significant association with ADHD and are predictors of ADHD in children. This finding is alike with that reported by Rothen et al. [30]. In general, it seems that the maternal somatic or psychiatric disorders during pregnancy in addition to providing the biological backgrounds for psychiatric disorders in children may disturb the nurturing role of parents and consequently result in undesirable conditions for family members.

According to our findings, trauma to the abdomen during pregnancy (severe enough to be focus of clinical attention) may be predictive of ADHD in children. In the present study, we considered the general history of trauma during pregnancy and did not differentiate between different types of trauma with different severities. However, it seems that the type and severity of trauma and probable amount of injury are important factors in this regard, as some of studies have emphasized on the association of severe head trauma with ADHD [6]. Our findings again emphasize on the role of trauma in ADHD and indicate a need for more definite studies in this field.

Another part of findings in this study showed that frequency of some other pregnancy-related events, that is, unwanted pregnancy, abortion attempt, X-ray exposure, infections, dysentery, bleeding, and preeclampsia has no probable effect on development of ADHD.

Although the unwanted pregnancy and subsequently the attempted abortion are public and universal problems effective on women, families, and society [31], it seems that Iranian culture and religious beliefs about humanitarian values may be effective in psychological adjustment in women and families with unwanted pregnancy and inhibit the negative psychological effects of it in the development of ADHD symptoms in the children.

Based on our findings, in general, infections, dysentery, bleeding, and preeclampsia during pregnancy are not risk factors for ADHD; it seems that ADHD is a profound disorder, and the situations in which these factors develop may not have sufficient duration and severity to cause ADHD. Indeed these types of pregnancy-related factors may have different severities, and therapeutic interventions may inhibit real effects of these factors during pregnancy. Nevertheless, some studies have shown that the ADHD children's mothers compared with healthy children's mothers have more viral disease and rash [32]. To conclude more definite results in this regard, there is a need for more detailed studies regarding the type, duration, severity, and therapeutic interventions for these types of pregnancy-related factors.

The present study had some limitations among which the most important one was the nonrandom selection of the case group. This may lead to the likelihood of selection bias in case samples and make limitations regarding the generalizability of the results. On the other hand, as getting the information about pregnancy-related risk factors was mainly based on the history taking from mothers, the recall bias may also affect the results. However, the findings of our study emphasizes on the likelihood of influence of some environmental factors in development of ADHD. Each of these factors considered in our study as a risk factor needs to be tested and confirmed through next methodologically appropriate researches in this field.

## 5. Conclusion

According to our findings somatic and psychiatric disorders, alcohol and cigarette exposure, trauma to the abdomen during pregnancy and election of cesarean section for labor are some of risk factors for ADHD. However, it is difficult to explain the definite mechanism of effect of these pregnancy-related factors in development of ADHD in children. As mentioned earlier in this paper, genetic predisposition is a major cause in ADHD, but it does not follow the Mendelian patterns of inheritance, and it is likely to be a polygenetic disorder so that genes can exert their influence only via interaction with the environment. Pregnancy-related maternal factors may be considered as some of these environmental factors that exert their influence through interaction with genetic predisposition in the targeted child with ADHD.

## Figures and Tables

**Table 1:** *t-*test results for comparison of mean parental ages at pregnancy and pregnancy duration in case and control groups.

Variable	Group				
	ADHD	Control	*t*	df	*P* value
	(Mean ± SD)	(Mean ± SD)			
Maternal age at pregnancy (years)	25.60 ± 5.16	25.04 ± 4.95	1.01	328	0.32
Paternal age at pregnancy (years)	30.99 ± 5.40	30.20 ± 6.26	1.22	328	0.22
Pregnancy duration (months)	8.90 ± 0.45	8.94 ± 0.37	0.96	328	0.34

**Table 2:** Frequency distribution (percent) of pregnancy-related factors among case and control groups.

Variables	Group	Yes	No	OR (95% CI)	Fisher's exact test	*P* value
Somatic disease	ADHD	21 (12.8%)	143 (87.2%)	3.91 (1.5–9.9)	*χ* ^2^ = 9.27	0.002
	Control	6 (3.7%)	160 (96.4%)			

Psychiatric disorder	ADHD	27 (16.5%)	137 (83.5%)	16.1 (3.7–69.1)	*χ* ^2^ = 23.96	0.001
	Control	2 (1.2%)	164 (98.8%)			

Alcohol and cigarette exposure	ADHD	15 (9.1%)	149 (90.9%)	4.1 (1.3–12.5)	*χ* ^2^ = 6.9	0.009
	Control	4 (2.4%)	162 (97.6%)			

Cesarean section	ADHD	103 (62.8%)	61 (37.2%)	3.0 (1.9–4.7)	*χ* ^2^ = 24.53	0.001
	Control	59 (35.5%)	107 (64.5%)			

Trauma to the abdomen	ADHD	8 (4.9%)	156 (95.1%)		*χ* ^2^ = 2.41	0.14
	Control	3 (1.8%)	163 (98.2%)			

X-ray exposure	ADHD	3 (1.8%)	161 (98.2%)		*χ* ^2^ = 1.03	0.37
	Control	1 (0.06%)	165 (99.4%)			

Infections	ADHD	3 (1.8%)	161 (98.2%)		*χ* ^2^ = 0.13	1.000
	Control	4 (2.4%)	162 (97.6%)			

Dysentery	ADHD	0 (0%)	164 (100%)		*χ* ^2^ = 4.0	0.12
	Control	4 (2.4%)	162 (97.6%)			

Bleeding	ADHD	6 (3.7%)	158 (96.3%)		*χ* ^2^ = 0.44	0.54
	Control	4 (2.4%)	162 (97.6%)			

Preeclampsia	ADHD	6 (3.7%)	158 (96.3%)		*χ* ^2^ = 0.27	0.78
	Control	8 (4.9%)	158 (96.3%)			

Unwanted pregnancy	ADHD	25 (15.2%)	139 (83.7%)		*χ* ^2^ = 0.06	0.88
	Control	27 (16.3%)	139 (83.7%)			

Attempted abortion	ADHD	3 (1.8%)	161 (98.2%)		*χ* ^2^ = 1.6	0.33
	Control	7 (4.2%)	159 (95.8%)			
